# Phasic and Tonic Locus Coeruleus Stimulation Associated Valence Learning Engages Distinct Adrenoceptors in the Rat Basolateral Amygdala

**DOI:** 10.3389/fncel.2022.886803

**Published:** 2022-05-09

**Authors:** Tamunotonye Omoluabi, Kyron D. Power, Tayebeh Sepahvand, Qi Yuan

**Affiliations:** Division of Biomedical Sciences, Faculty of Medicine, Memorial University of Newfoundland, St. John’s, NL, Canada

**Keywords:** locus coeruleus, phasic, tonic, olfactory, valence, amygdala

## Abstract

Reward exploitation and aversion are mediated in part by the locus coeruleus (LC), a brainstem structure significantly involved in learning and memory via the release of norepinephrine. Different LC firing patterns are associated with different functions. Previously, we have shown that high tonic and phasic LC activation signal negative and positive valence, respectively, via basolateral amygdala (BLA) circuitry. Tonic LC activation is associated preferentially with BLA-central amygdala (CeA) activation, while phasic LC stimulation preferentially recruits the BLA-nucleus accumbens (NAc) pathway. Here, we ask if phasic and tonic LC activation-associated valence learning requires different adrenoceptors in the BLA, in comparison with the odor valence learning induced by natural reward and aversive conditioning. Using optogenetic activation of the LC and local drug infusions in the BLA, we show that phasic LC activation-induced positive odor valence learning is dependent on both α_*1*_ and β-adrenoceptors, whereas tonic LC activation induced-negative odor valence learning depends on β-adrenoceptors only. In parallel, both α_*1*_ and β-adrenoceptors were required in the odor valence learning induced by reward while aversive conditioning was dependent on β-adrenoceptors. Phasic stimulation and reward conditioning likewise activated more NAc-projectors of the BLA, in comparison to tonic and aversive conditioning. There was a higher proportion of α_1_^+^ cells in the NAc-projectors compared to CeA-projectors in the BLA. Together, these results provide insight into the mechanisms underlying the effects of tonic and phasic activation of the LC, and more generally, negative and positive valence signaling.

## Introduction

The locus coeruleus (LC), a brainstem neuromodulatory nucleus, is the brain’s main source of norepinephrine (NE; Loizou, 1969). With widespread projections throughout the brain, the LC-NE system is well-structured to exert global effects through the ubiquitous release of NE (Aston-Jones and Cohen, 2005). A key component of the stress response, the LC signals arousal and is implicated in cognitive processes such as learning and memory, the flipside of which is its involvement in disorders of learning and memory, such as Alzheimer’s disease (Berridge and Waterhouse, 2003; Sara, 2009; Braak et al., 2011; Mather et al., 2016; Mather and Harley, 2016; Omoluabi et al., 2021). The LC exhibits two fundamental modes of activity, phasic and tonic, which adjust levels of NE at projection sites (Aston-Jones and Cohen, 2005). Typically, phasic LC activity occurs in response to salient stimuli, functioning to facilitate external and internal responses to such stimuli (Aston-Jones and Cohen, 2005; Bouret and Sara, 2005; Mather et al., 2016). Tonic activation on the other hand, fluctuates with general arousal levels, with lowest activity occurring during sleep, and highest activity during periods of high stress (Aston-Jones and Bloom, 1981; Carter et al., 2010). NE exerts its effects at target sites through its interaction with adrenoceptors, which vary in affinity from high affinity α_2_- to lower affinity α_1_- and β subtypes (Arnsten, 2000). Theories of LC function emphasize its role in altering the gain of activation at target sites to subserve functions such as behavioral adaptation and efficient responding to salient stimuli at both short- and long-term time scales (Aston-Jones and Cohen, 2005; Bouret and Sara, 2005; Mather et al., 2016). More recently, the idea of an anatomically and functionally modular LC extends these theories by proposing the additional possibility of point-to-point communication by way of functionally distinct LC nodes (Chandler et al., 2014, 2019; Uematsu et al., 2017; Poe et al., 2020).

Recent work suggests that distinct recruitment in the same target structure by different LC temporal activation patterns (phasic vs. tonic) can lead to divergent downstream circuitry activation associated with diverse behavior (McCall et al., 2017; Ghosh et al., 2021; Harley and Yuan, 2021). For instance, we have shown that, through its distinct activation modes, global activation of the LC can recruit different subpopulations of the basolateral amygdala (BLA), an effect which may be mediated by a heterogeneous distribution of adrenoceptors across BLA subpopulations (Ghosh et al., 2021). Furthermore, we have shown that these activation patterns can signal opposing valences, an effect which requires BLA involvement (Ghosh et al., 2021). Here, we sought to dissect the roles of specific adrenoceptors of the BLA in these findings and further to relate these processes to those occurring in naturally produced valence. To address this, we firstly characterized the contribution of various adrenoceptors in the valence effects elicited by LC photo-activation and natural reward- and aversive learning. We then used retrograde tracing to compare the recruitment of reward circuitry in the BLA during artificially and naturally produced valence. Finally, we used immunohistochemistry to study differences in adrenoceptor distribution across BLA subpopulations.

## Materials and Methods

### Subject and Design

For valence learning with LC photo-activation, offspring of homozygous tyrosine hydroxylase (TH)-Cre male breeders (Horizon) and Sprague Dawley (SD) female breeders (Charles River) of both sexes were used. For valence learning with natural stimuli, SD rats were used. All rats were housed in a 12-h light/dark cycle (7 am–7 pm light cycle) with *ad libitum* access to food and water, except during food deprivation (20 g food per day). All experimental protocols followed the guidelines of the Canadian Association for Animal Care and were approved by the Memorial University of Newfoundland Animal Care Committee.

### Viral Transduction and Stereotaxic Surgery

Animals were anesthetized under isoflurane in an induction chamber and transferred to a stereotaxic frame. AAVdj-EF1a-DIO-hChR2 (H134R)-mCherry (Neurophotonics) was bilaterally infused in the LC (AP: −11.8 to 12.2 mm, ML 1.2 and 1.4 mm, and DV 6.3 mm from bregma) of 5–6 month old TH-Cre rats. The infusion cannula was lowered at an angle of 20° to avoid the sigmoid sinus (Ghosh et al., 2019, 2021). The virus was infused at a rate of 0.5 μl/min with a total volume of 0.7 μl consisting of fluorescent beads and virus in a ratio of 2:5 (Ghosh et al., 2021). Two months following virus infusion, an optical cannula (Doric Lenses) was implanted in the LC using the same coordinates.

Basolateral amygdala cannula surgery was either carried out alone in SD rats or together with LC cannula implantation in TH-CRE rats. Twenty-three gauge metal guide cannulas were implanted bilaterally (AP: −2.5 mm, ML: 4.9 mm, and DV: 7.8 mm from bregma) (Ghosh et al., 2021). Animals were allowed to recover for at least 1 week before the onset of behavioral experiments.

For retrograde tracing experiments, Cholera Toxin subunit B (CTB) was infused bilaterally in the nucleus accumbens (NAc; 200 nL; AP: +1 mm, ML: 1 mm bilateral, and DV: 6.5 mm from bregma) or central amygdala (CeA; 150 nL; AP: −2.1 mm, ML: 4.2 mm, and DV: 7.5 mm from bregma). CTB-594 or CTB-488 (1% w/v in phosphate buffer; Invitrogen) was infused by a 32 g beveled 1 μL Hamilton syringes (Neuros 7001 KH) attached to a vertical infusion pump (Pump 11 Elite; Harvard Apparatus). Each infusion lasted 5 min, followed by a 5-min wait before withdrawing the syringe. Rats were allowed 1–2 weeks for recovery before the behavioral experiments or perfusion with 4% paraformaldehyde (PFA).

### Behavioral Experiments

#### Drug Infusion

Adrenoceptor antagonist infusions were conducted during valence learning. The α_1_ adrenoceptor antagonist prazosin (Sigma) and β_1/2_ adrenoceptor antagonist propranolol (Sigma) were infused in the BLA (2.5 μg/0.5 μl per hemisphere) (Ferry et al., 1999b; Giustino et al., 2020). Control rats received saline infusions. Infusions took place 20–30 min before training at 0.25 or 0.5 μl/min, through stainless steel 33-gauge internal cannula connected to a 10 μl Hamilton syringe by polyethylene tubing and driven by a Pump 11 Elite Syringe pump (Harvard Apparatus). The internal cannula extended 1 mm below the guide cannula in the BLA. The infusion cannula was left in place for an additional 1 min to allow for diffusion. All drugs used were freshly prepared.

#### Odor Conditioning With Locus Coeruleus Light Stimulation

Bilateral photo-activation of the LC was delivered via two laser light sources (LDFLS_450; Doric Lenses) at 450 nm (20 mW/mm^2^ at fiber tip) through mono-fiber optic patch cords. Ten hertz phasic (300 ms every 2 s; light pulse duration 30 ms) or 25 Hz tonic stimulation patterns were delivered using Doric software (Ghosh et al., 2021). We have previously shown that light stimulation (10–30 Hz) generated ∼8–15 Hz output in LC neurons in anesthetized rats (Ghosh et al., 2021; Omoluabi et al., 2021), which are in the range of LC physiological activation [see discussion in Ghosh et al. (2021)].

In a conditioned odor preference test (COPT; Ghosh et al., 2021), TH-Cre rats were first habituated to a T-maze (long arm 183 cm × 19 cm; neutral arm 19 cm × 19 cm; 20.5 cm high wall) for two consecutive days. On the morning of day 3, two sponges infused with 60 μl of two odors (O1 and O2) were placed in the T-maze’s left and right arms, respectively, and the rats were allowed to explore both sponges freely for 10 min. Time spent on both arms was manually recorded and videotaped for later analysis. The same test was repeated in the afternoon with the sponges swapped in the two arms. Morning and afternoon sessions started at 9 am and 1 pm, respectively.

On day 4, local infusion of vehicle, prazosin, or propranolol in the BLA occurred at 20–30 min before training. In the morning session, rats were confined to one arm with O1. Rats received either 10 Hz phasic or 25 Hz tonic stimulation of the LC for 10 min (Ghosh et al., 2021). In the afternoon session of the same day, animals were confined to the same arm with O2 and no light stimulation. On day 5, rats were confined to the other arm and received the same treatment as on day 4. Tonic stimulated rats were tested for odor preference on day 6, while phasic stimulated rats continued 2 more rounds of training (6 days total) before testing.

On test day, rats freely explored both sponges in the morning and afternoon sessions without photo-activation. Arm time was video recorded, and preference score was calculated as the ratio of time in the O1 arm to the total time in both arms.

#### Odor Reward Conditioning

In a modified food retrieval test (Ghosh et al., 2021), rats were food-deprived for 4–7 days before training and food deprivation continued throughout the experiments. Following a 3–5-day habituation to the training chamber (a 60 cm × 60 cm × 40.5 cm Plexiglas box) and sponge with a food pellet (chocolate cereal), rats performed odor discrimination learning consisting of 16 trials/day for 3 days. Two sponges were infused with 60 μl of either O1 or O2. A retrievable chocolate cereal was placed in a 2 cm hole on the surface of the O1 sponge, while a non-retrievable cereal was placed in a hidden hole in the O2 sponge to control for the smell of chocolate cereal. During the trial, rats freely explored the box and sponges; the position of the sponges was changed in each trial. A trial ended when a nose poke was made in the sponge, irrespective of the sponge choice. A trial was termed correct response if a nose poke was made in the O1 sponge containing the retrievable cereal and the food was retrieved. Rats were confined to a corner in the test box with a barrier for 20 s between the trials. The percentage of correct responses was calculated as the number of correct nose pokes over the total number of nose pokes. A trial ended if no nose poke occurred in 3 min and was excluded from analysis. In the control condition, the food pellet was paired with both O1 and O2 pseudo-randomly.

#### Odor Aversive Conditioning

Rats were habituated to a shock chamber (San Diego Instruments, San Diego, CA, United States) for 30 min for three consecutive days. On the fourth day, rats were exposed to four trials of shock paired with an odor at the 5th, 15th, 20th, and 30th minute during a 30 min training session. An odor was delivered to the shock chamber by an olfactometer for 1 min at each time point, terminating with the shock (0.5 mA for 1 s). On the fifth day (test day), rats were exposed to the shock chamber with no odor delivery for 5 min to measure baseline activity, followed by a 5 min exposure to the conditioned odor. The experiment was videotaped and the percentage of time freezing (freezing defined as no body movement except breathing) was calculated. Rats exposed to only odor were used as control.

#### Odorants Used in the Behavioral Experiments

For odor conditioning with photo-activation, three pairs of odorants were used: vanilla (2%) vs. peppermint (2%); almond (2%) vs. coconut (2%); benzaldehyde (0.05%) vs. isoamyl acetate (0.05%) (Ghosh et al., 2021). For odor food reward conditioning, almond/coconut or vanilla/peppermint pairs were used. For odor shock conditioning, either benzaldehyde or vanilla was used. Rats were trained in multiple experiments (vehicle or drugs) with different odors in each experiment.

#### cFos Induction

For odor-LC photo-activation conditioning, TH-Cre rats were habituated in a room in their home cages for 2 days. A 2-day, 10 min LC photo-activation of either 10 Hz phasic or 25 Hz tonic were carried out with exposure to an odorized sponge (benzaldehyde) in the home cage. Ninety minutes after odor exposure and LC photo-activation, rats underwent transcardiac perfusion with 4% PFA. For cFos induction following natural odor conditioning, rats were re-exposed to the conditioned odor (almond) in the home cage for 10 min, 24 h following the odor preference test or freezing test, and perfused 90 min later with 4% PFA. Brains were extracted and stored in 4% PFA solution overnight and then transferred to PVP solution (1% polyvinylpyrrolidone, 30% sucrose, 30% ethylene glycol in 0.1 M PBS) storage solution until used for IHC.

### Immunohistochemistry

Free floating sections of 50 μm were cut using a compresstome (Precision Instrument). Sections were washed 5 min twice in Tris (0.1 M, pH 7.6) buffer. This was followed by a 10 min wash in Tris A (0.1% Triton X in Tris buffer) and then in Tris B (0.1% Triton X and 0.005% BSA in Tris buffer) before blocking with 10% normal goat serum (Sigma-Aldrich, Oakville, ON, Canada) for 1.5 h. Sections were then transferred and washed in Tris A and then Tris B for 10 min each. This was followed by incubation in a primary antibody prepared in Tris B [cFos, 1:2000, Cell Signaling; α_1_, 1:2000, Alomone; β_1_, 1:2000, Abcam; β_2_, 1:2000, Alomone; Dopamine beta-hydroxylase (DBH), 1:2000, Millipore-Sigma] at 4°C for two nights. The sections were then washed 10 min each in Tris A and Tris B, followed by incubation in a secondary antibody (anti-rabbit Alexa 647, 1:1000, Thermo Fisher Scientific) prepared in Tris B for 40 min and then a 10 min wash in Tris buffer. Sections were mounted onto slides, and coverslipped with DAPI mounting media.

### Imaging and Analysis

Fluorescence images were captured using an EVOS 5000 (Thermo Fisher Scientific, Mississauga, ON, Canada) microscope. Images were analyzed using ImageJ software. For cFos activation, images underwent background subtraction before automatic cell counting using the Trainable Weka Segmentation plugin. For adrenoceptor distribution, images underwent background subtraction before manual cell counting. Three images per animal through the same rostral to caudal range were analyzed.

### Statistics

**FIGURE 1:**
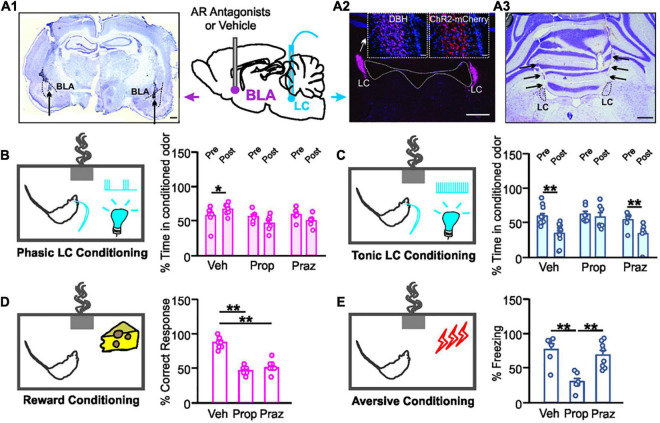
Distinct adrenoceptor requirement for odor valence learning. **(A1)** Example image of bilateral basolateral amygdala (BLA) cannula implantation targeting. Dashed lines outline BLAs. Arrows indicate cannula tips. Scale bar, 500 μm. **(A2)** ChR2-expressing mCherry cells in the locus coeruleus (LC) co-localize with dopamine beta-hydroxylase (DBH) positive cells. Scale bars, 500 μm and 50 μm (insets). **(A3)** Example image of bilateral optical cannula tracks targeting LCs (black arrows). Scale bar, 500 μm. **(B)** Time spent in the conditioned odor pre- and post- odor associative training with 10 Hz phasic LC photo-activation. **(C)** Time spent in the conditioned odor pre- and post- odor associative training with 25 Hz tonic LC stimulation. **(D)** Percentage correct response toward the rewarded odor during the testing. **(E)** Percentage time spent freezing upon the odor exposure during the testing. Veh, vehicle; Prop, propranolol; Praz, prazosin. *p < 0.05, **p < 0.01.

**FIGURE 2:**
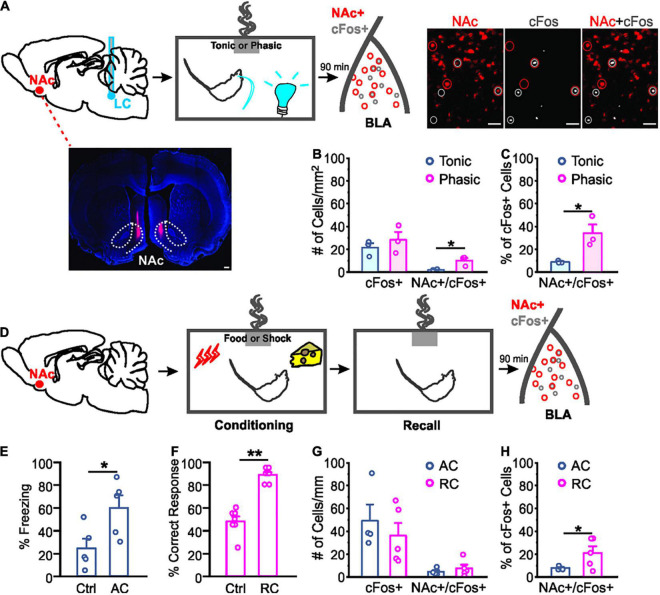
Different activation of nucleus accumbens projecting neurons in the basolateral amygdala by odor valence learning. **(A)** Schematics of the odor conditioning with locus coeruleus (LC) stimulation in rats with CTB injected in the nucleus accumbens (NAc; dotted lines outline NAc core and shell). Example images of NAc CTB (lower left, scale bar: 500 μm) and cFos staining (right, scale bars: 50 μm) are included. **(B)** Numbers of cFos^+^ cells and NAc-projecting cFos^+^ cells per mm^2^ following odor conditioning with light stimulation. **(C)** Percentage NAc-projecting cells in total active cells (cFos^+^) following odor conditioning with photo-activation. **(D)** Schematics of the odor conditioning with natural stimuli (food or shock). **(E)** Percentage time spent freezing upon the odor exposure during the testing in odor conditioned rats with shock. **(F)** Percentage correct response toward the rewarded odor during the testing in odor conditioned rats with food reward. **(G)** Numbers of cFos^+^ cells and NAc-projecting cFos^+^ cells per mm^2^ following different valence odor conditioning. **(H)** Percentage NAc-projecting cells in total active (cFos^+^) cells. AC, aversive conditioning; RC, reward conditioning. *p < 0.05, **p < 0.01.

**FIGURE 3:**
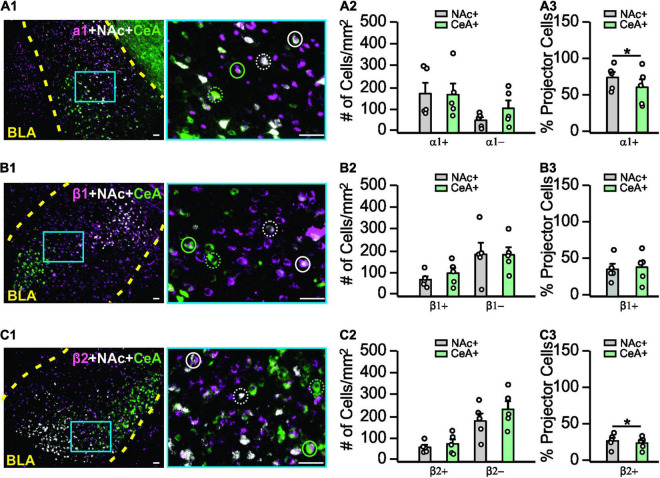
Adrenoceptor subtype expressions in the basolateral amygdala. **(A1–A3)** Expression patterns of α_*1*_-adrenoceptors in the BLA and co-labeling with NAc- or CeA- projecting cells. **(A1)** Example image of α_*1*_-adrenoceptor staining (magenta), NAc- (white), and CeA- (green) projecting cells are indexed by CTB. Image on the right is the zoom in image of the area indicated by the blue square on the left image. Solid circles, α_1_^+^ projecting cells. Dashed circles, α_1_^–^ projecting cells. **(A2)** Numbers of α_1_^+^ and α_1_^–^ NAc- and CeA-projecting cells. **(A3)** Percentage of α_1_^+^ projecting neurons over the total projecting neurons. **(B1–B3)** Expression patterns of β_1_-adrenoceptors in the BLA and co-labeling with NAc or CeA projecting cells. Solid circles, β_1_^+^ projecting cells. Dashed circles, β_1_^–^ projecting cells. **(C1–C3)** Expression patterns of β_2_-adrenoceptors in the BLA and co-labeling with NAc- or CeA-projecting cells. Solid circles, β_2_^+^ projecting cells. Dashed circles, β_2_^–^ projecting cells. Scale bars: 50 μm. *p < 0.05.

Paired Samples *t*-Tests (two tailed) were used in Figures 1B,C for the pre- and post-conditioning comparison within groups. The critical level of significance was set at 0.05. One-way analyses of variance (ANOVA) followed by *post hoc* Tukey’s Tests were used in Figures 1D,E. Unpaired *t*-Tests were used in Figure 2. A Two-way repeated ANOVA was used to examine the overall patterns of adrenoceptor distribution in Figures 3A2–C2 and a paired *t*-test was used to compare percentages of NAc- and CeA-projecting cells that expressed adrenoceptors.

## Results

### Different Valence Learning Is Mediated by Distinct Adrenoceptors in the Basolateral Amygdala

We first explored the adrenoceptor requirement for odor valence learning induced by LC patterned stimulations. Rats underwent LC AAV infusions and BLA and LC cannula implantations (Figures 1A1–A3) before behavioral training. LC phasic photo-activation paired with the conditioned odor led to a preference to that odor in vehicle infused rats, indexed by a higher percentage time spent in the conditioned odor chamber post-training (*t*_6_ = 2.909, *n* = 7, *p* = 0.027; Figure 1B). Both the β-adrenoceptor antagonist propranolol (*t*_5_ = 1.69, *n* = 6, *p* = 0.153) and α_1_-adrenoceptor antagonist prazosin (*t*_5_ = 1.279, *n* = 6, *p* = 0.257) infusion in the BLA prevented the conditioned preference to the odor (Figure 1B). On the other hand, high tonic LC photo-activation induced odor avoidance following the odor-photo-activation conditioning in vehicle rats (*t*_11_ = 2.48, *n* = 12, *p* = 0.042; Figure 1C). β-adrenoceptor (*t*_6_ = 0.707, *n* = 7, *p* = 0.506), but not α_1_-adrenoceptor (*t*_5_ = 6.097, *n* = 6, *p* = 0.002) blockade in the BLA prevented aversive odor learning (Figure 1C). We have shown previously that light stimulation without ChR2 expression in LC neurons did not lead to either preference or avoidance of the conditioned odor (Ghosh et al., 2021).

Similarly, both propranolol and prazosin infusions in the BLA prevented odor preference formation using food as a reward [*F*_2.21_ = 59.010, *n* = 8/7/9 (saline/propranolol/prazosin), *p* < 0.01; Figure 1D]. Vehicle rats had significantly more correct nose pokes toward the food-rewarded odor sponge than either the propranolol or the prazosin group (*p* < 0.01; Figure 1D). In odor aversive conditioning with electrical shock, propranolol, but not prazosin, prevented the formation of odor aversion [*F*_2.18_ = 14.015, *n* = 6/7/8 (saline/propranolol/prazosin), *p* < 0.01; Figure 1E]. The propranolol group spent significantly less time freezing to the conditioned odor during the test than the other two groups (*p* < 0.01; Figure 1E).

Together, these results suggest that positive odor valence learning is mediated by both α_1_- and β-adrenoceptors in the BLA, whereas negative odor valence depends on BLA β-adrenoceptors.

### Positive Odor Valence Learning Activates a Larger Proportion of Nucleus Accumbens-Projecting Basolateral Amygdala Neurons Compared to Negative Valence Learning

We compared activated NAc-projecting cells (labeled by retrograde CTB tracer injected in the NAc) in the BLA following odor conditioning with different LC light patterns. Activated NAc-projectors of the BLA were indexed by cFos staining (Figure 2A). Even though the number of cFos^+^ cells were similar in phasic and tonic groups [*t*_4_ = 0.839, *n* = 3/3 (phasic/tonic), *p* = 0.448; Figure 2B], activated NAc-projecting cells (NAc^+^/cFos^+^) in the phasic group were more in number than the tonic group (*t*_4_ = 3.310, *p* = 0.030; Figure 2B), and occupied a higher percentage of total cFos^+^ cells in the BLA (*t*_4_ = 3.273, *p* = 0.031; Figure 2C).

We then compared odor-evoked cFos activation in the BLA following natural odor conditioning with either food or shock as the unconditioned stimulus. BLA neurons engaged in fear learning are re-activated during memory retrieval (Reijmers et al., 2007). Following successful conditioning, the shock-paired rats showed significantly more freezing to the conditioned odor compared to controls [*t*_8_ = 2.596, *n* = 5/5 (control/AC), *p* = 0.032; Figure 2E], whereas the food rewarded rats showed higher percentage of correct nose poke toward the food-rewarded odor sponge [*t*_12_ = 8.227, *n* = 7/7 (control/RC), *p* < 0.001; Figure 2F]. Similar to odor-photo-activation conditioning, total number of cFos^+^ cells were comparable in the aversive and reward learning groups [*t*_7_ = 0.744, *n* = 4/5 (AC/RC), *p* = 0.481; Figure 2G]. However, rats that underwent reward learning showed a higher percentage of NAc-projector activation upon odor re-exposure, compared to the rats that experienced shock training (*t*_7_ = 2.007, *p* = 0.042; Figure 2H).

### Adrenoceptor Subtype Distribution in the Basolateral Amygdala

We next explored the expression patterns of adrenoceptor subtypes (α_*1*_, β_*1*_, β_*2*_) in the BLA using immunohistochemistry. Co-labeling with NAc- and CeA-projectors was measured. We compared the numbers of the projector cells that expressed an adrenoceptor (co-labeled with an adrenoceptor) and those without adrenoceptor labeling (Figure 3). For the α_*1*_-adrenoceptor (Figures 3A1–A3), no difference in overall distribution were observed by 2-way repeated ANOVAs (*F*_1,4_ = 2.318, *n* = 5, *p* = 0.203; Figure 3A2). However, NAc-projectors have a higher proportion of α_1_^+^ cells (73.8 ± 7.38%) compared to CeA-projectors (60.59 ± 10.76%; *t*_4_ = 3.627, *p* = 0.022; Figure 3A3). For the β_1_-adrenoceptor (Figures 3B1–B3), there was no difference in the distribution (*F*_1,4_ = 5.009, *n* = 5, *p* = 0.089; Figure 3B2) and the proportion of β_1_^+^ cells in the projectors (*t*_4_ = 0.704, *p* = 0.520; Figure 3B3). β_2_-adrenoceptors (Figures 3C1–C3) showed no difference in overall pattern (*F*_1,4_ = 3.029, *n* = 5, *p* = 0.157; Figure 3C2), however, a larger proportion of β_2_^+^ cells was observed in NAc-projectors (27.95 ± 4.25% vs. 24.91 ± 3.76% CeA-projectors; *t*_4_ = 3.539, *p* = 0.024; Figure 3C3).

## Discussion

Our work demonstrates positive and negative valence signaling associated with phasic and tonic LC activity, respectively [see also Ghosh et al. (2021)]. This is in line with recent findings suggesting the role of tonic LC activity in negative valence signaling (McCall et al., 2015, 2017; Hirschberg et al., 2017; Llorca-Torralba et al., 2019), as well as findings of positive valence associated with LC activation (Chen et al., 2021). Synaptic plasticity in the BLA mediates the formation of associative memories of both positive and negative valences (Namburi et al., 2015). Recent work has suggested that the effect of tonic LC activity may be in part due to recruitment of CeA-projectors of the BLA (McCall et al., 2017; Ghosh et al., 2021). We have shown that LC tonic activation preferentially recruits CeA-projectors of the BLA and is able to produce real-time and conditioned odor aversion in rats (Ghosh et al., 2021). Furthermore, phasic LC activation can produce real-time and conditioned odor preference, possibly through the recruitment of NAc-projectors of the BLA (Ghosh et al., 2021). This body of evidence is in line with studies showing functional segregation of BLA subpopulations. Tye’s group has shown that selective activation of BLA NAc- and CeA- projectors produces positive and negative reinforcement, respectively (Namburi et al., 2015). Here we show that positive valence induced by either LC phasic activation or natural reward is associated with more activation of NAc-projectors in the BLA compared to LC tonic activation or aversive association, respectively. Thus, reward learning may convey positive valence *via* the LC-BLA-NAc pathway, while aversive learning may induce negative valence *via* the LC-BLA-CeA pathway, contingent on the patterns of the LC activation.

We have previously shown that the valence-producing effect of LC photo-activation patterns depends upon BLA adrenoceptor recruitment. Blocking both α- and β-adrenoceptors in the BLA prevented LC photo-activation-induced valence learning (Ghosh et al., 2021). How LC activation patterns engage distinct BLA sub-populations *via* differential adrenoceptor recruitment and lead to divergent downstream activation was not known. Here we demonstrate the unique requirement of adrenoceptor subtypes for LC photo-activation-induced valence. We showed that tonic LC stimulation requires β-adrenoceptor engagement to elicit negative valence, while phasic LC stimulation requires either β- or α_1_-adrenoceptors to elicit positive valence. Results from our natural learning paradigms closely reflected those from LC photo-activation. Aversive learning selectively required β-adrenoceptor engagement, while reward learning required engagement of β- and α_1_-adrenoceptors.

These findings are in line with the known role of BLA β-adrenoceptors in aversive memory formation (McGaugh et al., 1996; Bush et al., 2010; Johansen et al., 2014). The involvement of α-adrenoceptors in aversive conditioning is less clear. A facilitating effect of BLA α_1_-adrenoceptor on β-adrenoceptor-mediated inhibitory avoidance learning is thought to involve enhanced cAMP production (Ferry et al., 1999a,b), while antagonizing BLA α_1_-adrenoceptors alone does not affect auditory fear conditioning but facilitates fear extinction (Lucas et al., 2019). Terazosin, an α_1_-adrenoceptor antagonist, facilitates fear conditioning and long-term potentiation *via* its effect on inhibitory neurons (Lazzaro et al., 2010). Opposing effects of β- and α_2_-adrenoceptors on NE and LC stimulation-induced BLA neuronal responses have been reported, with strong inhibitory effects on neuronal firing mediated by α_2_-, and milder excitatory effects mediated by β-adrenoceptors (Buffalari and Grace, 2007). Consistent with an inhibitory role, blocking α_2_-adrenoceptors in the BLA enhances avoidance memory retention (Ferry and McGaugh, 2008).

The roles of adrenoceptors in reward/appetitive learning are much less understood. The hedonic value of LC-NE was first proposed in the 70’s (Ritter and Stein, 1973) and has been supported by recent evidence (Chen et al., 2021; Ghosh et al., 2021). Our work provides some of the first evidence of specific BLA adrenoceptor involvement in positive valence formation.

Our results suggest that naturally encountered valence draws upon the same circuitry within the LC-BLA pathway as does our photo-activation-induced valence. The modes and amount of NE release associated with LC activation patterns may be critical in target structure neuronal recruitment such as in the BLA. It has been demonstrated that phasic LC stimulation releases higher amounts of NE in the prefrontal cortex than tonic stimulation, when stimulation pulse numbers are matched (Florin-Lechner et al., 1996). However, if the numbers of pulses are not matched and tonic stimulation yields a higher number of pulses per unit time (in our case for example), tonic stimulation likely causes higher NE release. One recent report using pupillometry (measuring changes in pupil diameter as a proxy for NE release) showed that continuous tonic stimulation for 10 s dilates pupils more than a short burst at a higher frequency (Privitera et al., 2020). While the complex relationship between firing pattern and NE release needs to be established in the future, our results nevertheless suggests that differential NE release by LC activation patterns is involved in distinct sub-population recruitment in the BLA. Results from immunohistochemistry showing higher number of α_1_^+^ cells within NAc-projectors of the BLA support a differential recruitment hypothesis. However, technical limitations elude precise measurement of adrenoceptor expression levels in BLA subpopulations. Fluorescence-activated cell sorting of specific neuronal populations (e.g., NAc-projecting vs. CeA-projecting) and qualitative protein or mRNA measurement, together with opto- or chemogenetic antagonism or activation of specific adrenoceptors in selective neuronal populations (Airan et al., 2009) may shed further light on the relationship between LC activation pattern, NE release and adrenoceptor recruitment in the BLA.

## Data Availability Statement

The original contributions presented in the study are included in the article/supplementary material, further inquiries can be directed to the corresponding author.

## Ethics Statement

The animal study was reviewed and approved by the Animal Care Committee of Memorial University of Newfoundland.

## Author Contributions

QY conceptualized the study. TO, KP, and QY designed the experiments and wrote the manuscript. TO, KP, and TS conducted the experiments. TO, KP, TS, and QY analyzed the data. All authors contributed to the article and approved the submitted version.

## Conflict of Interest

The authors declare that the research was conducted in the absence of any commercial or financial relationships that could be construed as a potential conflict of interest.

## Publisher’s Note

All claims expressed in this article are solely those of the authors and do not necessarily represent those of their affiliated organizations, or those of the publisher, the editors and the reviewers. Any product that may be evaluated in this article, or claim that may be made by its manufacturer, is not guaranteed or endorsed by the publisher.
